# Quantification of the effects of fur, fur color, and velocity on Time-Of-Flight technology in dairy production

**DOI:** 10.1186/s40064-015-0903-0

**Published:** 2015-03-26

**Authors:** Jennifer Salau, Ulrike Bauer, Jan H Haas, Georg Thaller, Jan Harms, Wolfgang Junge

**Affiliations:** Institute of Animal Breeding & Husbandry of Christian - Albrechts - University, Olshausenstraße 40, Kiel, 24098 Germany; Institute for Agricultural Engineering & Animal Husbandry of Bavarian State Research Center for Agriculture, Prof.-Dürrwaechter-Platz 2, Poing-Grub, 85586 Germany

**Keywords:** Dairy cow, Automated monitoring, Time-of-flight, Image processing, Fur color

## Abstract

With increasing herd sizes, camera based monitoring solutions rise in importance. 3D cameras, for example Time-Of-Flight (TOF) cameras, measure depth information. These additional information (3D data) could be beneficial for monitoring in dairy production. In previous studies regarding TOF technology, only standing cows were recorded to avoid motion artifacts. Therefore, necessary conditions for a TOF camera application in dairy cows are examined in this study. For this purpose, two cow models with plaster and fur surface, respectively, were recorded at four controlled velocities to quantify the effects of movement, fur color, and fur. Comparison criteria concerning image usability, pixel-wise deviation, and precision in coordinate determination were defined. Fur and fur color showed large effects (*η*^2^=0.235 and *η*^2^=0.472, respectively), which became even more considerable when the models were moving. The velocity of recorded animals must therefore be controlled when using TOF cameras. As another main result, body parts which lie in the middle of the cow model’s back can be determined neglecting the effect of velocity or fur. With this in mind, further studies may obtain sound results using TOF technology in dairy production.

**Electronic supplementary material:**

The online version of this article (doi:10.1186/s40064-015-0903-0) contains supplementary material, which is available to authorized users.

## Background

Multi-disciplinary approaches and technological solutions will be characterizing concepts in the agricultural science of the next decade. Especially solutions to monitor animal health in terms of body condition changes and lameness gain more and more importance, as these are meaningful issues in herd health management and herd productivity (Collard et al. 2000; Booth et al. 2004). There have been several camera-based studies during the last years reaching high rates of correct classification in lameness detection. In (Song et al. 2008; Pluk et al. 2012), and (Poursaberi et al. 2011) walking cows had been recorded using digital 2D cameras in side view position and methods concerning hooves, legs’ angles, and back posture came to use in the evaluation of cows’ gait, respectively. Moreover, various 2D-camera-based studies on automated body condition scoring have been presented. In (Azzaro et al. 2011) cow shapes were reconstructed using linear and polynomial kernel principal component analysis and the body condition score (BCS) was estimated. BCS prediction models based on five anatomical points were presented in (Bercovich et al. 2012).

Segmentation is always a difficult part of preprocessing when 2D digital images are used (Hertem et al. 2013), because changes in light conditions and scenery affect segmentation algorithms and complicate the definition of a common image background for all pictures. For this reason thermal images were considered for BCS determination in (Halachmi et al. 2013). BCS was assessed by fitting a parabola to the cow shape and full automation was reached. 3D cameras are another approach to overcome segmentation problems. As the pixel’s relative distances from the camera are known, the separation between fore- and background is easier. Furthermore, the usage of 2D data forces the projection of a threedimensional scenery onto a plane. Objects and their movement through threedimensional space can only be described accurately when spatial anomalies like distances diagonal or parallel to the camera’s line of sight are considered. Consequently, in (Krukowski 2009) images from a Time-Of-Flight (TOF) 3D camera were analyzed with regard to BCS determination. The rear view of dairy cows in standstill was recorded with a manually guided camera. In (Salau et al. 2014) and (Weber et al. 2014) a TOF-based system with automated calibration, animal identification and body trait gathering was introduced. The study was able to estimate the backfat thickness (BFT) using the characteristics extracted from the depth images. A different type of 3D camera was used in (Viazzi et al. 2014) and (Hertem et al. 2014). The Microsoft Kinect sensor (Microsoft 2010) works with the 3D measurement principle “Structured Light” (Fofi et al. 2004). The Kinect was used for lameness detection via back’s posture extraction in (Viazzi et al. 2014) and algorithms and results were compared to those obtained from 2D video recordings as presented in (Poursaberi et al. 2011). As the Kinect camera’s usage turned out to be promising, in (Hertem et al. 2014) the algorithm was improved and the classification performance was optimized.

Digital cameras are prone to error when used outdoors or in barn environment, because of sunlight conditions, dirt, fur-covered surfaces, and the animals’ movement. For a successful application of 3D cameras in monitoring solutions, their sensitivity towards fur, different fur colors, and animal movement should be analyzed. During data collection for (Weber et al. 2014) was found that fur and fur color changes cause imprecise TOF depth measurements. In addition, evaluations needed to be restricted to recordings of standing cows, as motion artifacts occurred. The dependence on the projected infrared pattern causes some limitations of “Structured Light” depth measurement for its part. Depth values can only be calculated from constellations of light dots not from a single dot, which causes difficulties in measuring thin objects (Lau 2013). Additionally, no depth value can be calculated between the light dots, which leads to a coarser depth resolution with increasing distance from the camera (Andersen et al. 2012). Furthermore, (Hansard et al. 2012) stated, that material properties strongly correlated to depth accuracy, and that both measurement principles had difficulties with various surfaces. This study would not compare the capabilities of Kinect and TOF depth sensors, because there have been detailed publications on this (i.e. (Langmann et al. 2012) where a TOF camera with a sensor similar to that used in SR4K was studied). The next generation (Microsoft 2014) of the Microsoft Kinect depth sensor is indeed a TOF camera. It has not been available for data collection during this study. Therefore, the present study quantified quality loss due to fur (color) and movement concerning TOF camera recordings. Indoor recordings of cow models were used to eliminate the effect of sunlight, and the software described in (Salau et al. 2014) was applied to them. The aim was to create a basis for a TOF camera application in moving dairy cows.

## Results

All the criteria showed the same differences and significant effects, independently of whether they were extracted from the original or the mirrored images (for explanations on the mirroring see section ‘Material and methods’, ‘Comparison criteria and statistical methods’). Only the data extracted from the original images is presented.

### Proportion of high quality images

As the ratio of high quality images (*N*_velocitiy_) to recorded images (*C*_velocitiy_), the HQIratio (section ‘Material and methods’, ‘Comparison criteria and statistical methods’, *Proportion of high quality images*) served as a measure for the usability of the recorded images:

(1) $$ \text{HQIratio}_{\text{velocitiy}}=\frac{N_{\text{velocitiy}}}{C_{\text{velocitiy}}}.  $$

Table 1 presents the numbers of recordings, the numbers of images that passed the quality tests, and the HQIratios for both models and all velocities.

**Table 1:** **The numbers of recorded images (** ***C***_***velocitiy***_ **), the number of images that passed all quality tests that had been integrated in the developed software (** ***N***_***velocitiy***_ **), and the ratios** $\boldsymbol {\text {HQIratio}_{\text {velocitiy}}=\frac {N_{\text {velocitiy}}}{C_{\text {velocitiy}}}}$ **for the plaster cast as well as the fur-covered model and all velocities**

	**Plaster cast**			**Fur-covered model**		
**Velocity**	***C***_**velocitiy**_	***N***_**velocitiy**_	**HQIratio**_**velocitiy**_	***C***_**velocitiy**_	***N***_**velocitiy**_	**HQIratio**_**velocitiy**_
Standstill	2155	1882	0.87	2138	2138	1.00
10 cm/s	542	371	0.68	411	141	0.34
20 cm/s	482	321	0.66	356	21	0.06
30 cm/s	370	231	0.62	267	4	0.015

**Figure 1:**
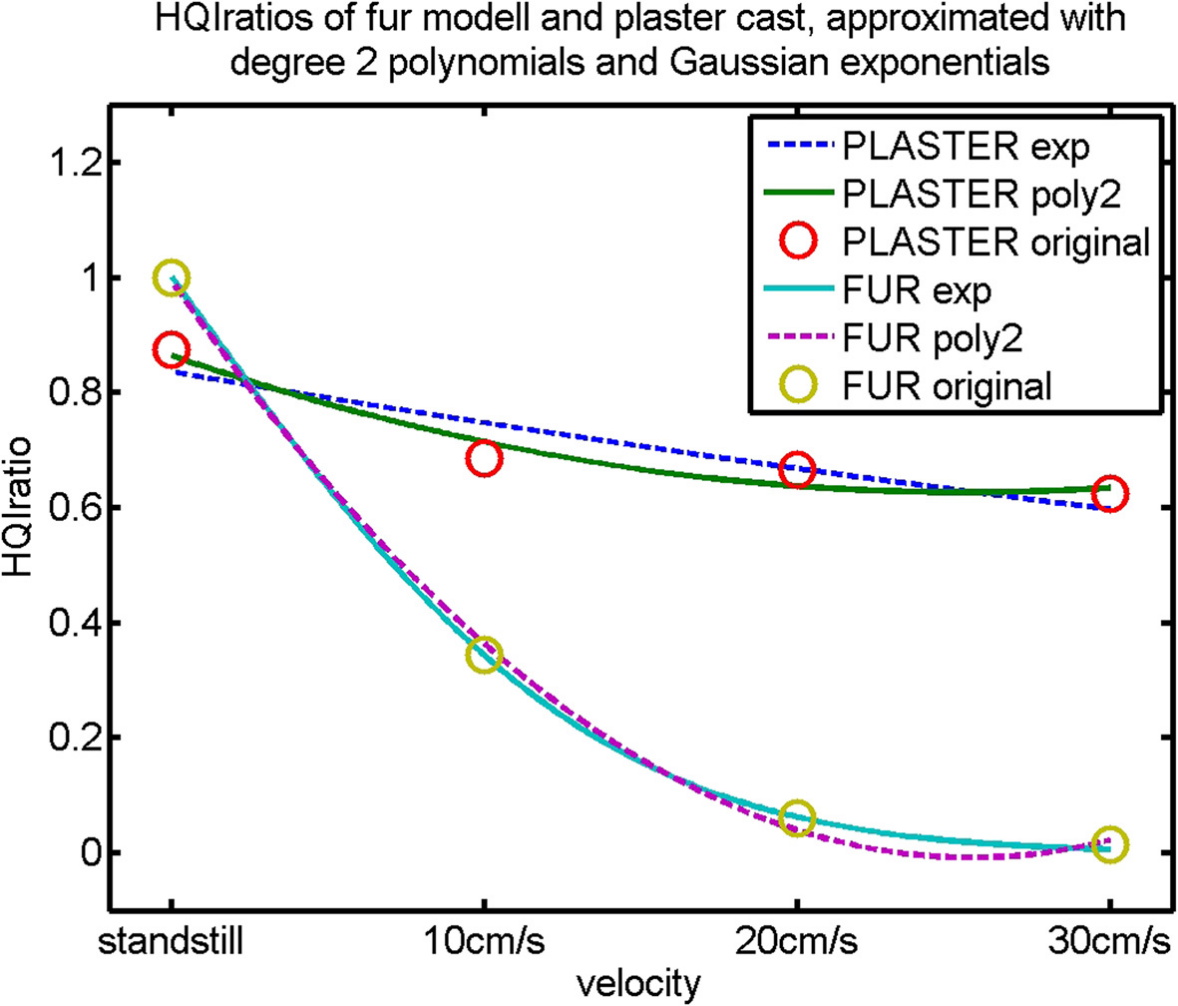
**Behavior of HQIratio with increasing velocity in comparison between models.** HQIratio is the quotient of the number of high quality images to the number of recorded images. The circles belong to the actual HQIratio values (olive: fur-covered model, red: plaster cast). For both models two types of functions have been fitted to the original values in a least square sense: a polynomial of degree two (purple: fur-covered model, green: plaster cast) and a Gaussian exponential function (cyan: fur-covered model, blue: plaster cast). The approximation that showed less goodness-of-fit is illustrated as dotted line, respectively.

For the plaster cast, the most significant decrease happened during the transition from standstill to movement, where the HQIratio dropped by ≈22% from 0.87 to 0.68. With the acceleration from 10 cm/s to 20 cm/s HQIratio dropped from 0.68 to 0.66, which was a decrease of ≈3%. In comparison with the final velocity of 30 cm/s, HQIratio fell by additional ≈6% to 0.62. The HQIratios of the fur-covered model dropped by 66% when the model started to move. The acceleration afterwards caused additional decreases in HQIratio by ≈82% (from 0.34 to 0.06), when speeding up from 10 cm/s to 20 cm/s, and 75% (from 0.06 to 0.015), when the final velocity of 30 cm/s was set. The polynomials of degree 2 (P _plaster_, P _fur_) and the Gaussian exponential functions (g _plaster_, g _fur_) that fit the vectors (HQIratio_0_, HQIratio_10_, HQIratio_20_, HQIratio_30_) best in a least square sense are given by

(2) $$\begin{array}{*{20}l} P_{\text{plaster}}(x) &= 0.037*x^{2} -0.261*x+ 1.087,\\ &\quad(\text{RMSD}=0.0432, \mathrm{R}^{2}=0.95), \end{array} $$

(3) $$\begin{array}{*{20}l} P_{\text{fur}}(x) &= 0.153*x^{2}-1.09*x+1.93, \\ &\quad(\text{RMSD}=0.0297, \mathrm{R}^{2}=0.99), \end{array} $$

(4) $$\begin{array}{*{20}l} g_{\text{plaster}}(x) &= 27.75*10^{29}*exp\left(-\left(\frac{x+1252.1 }{149.69}\right)^{2}\right),\\ &\quad(\text{RMSD}=0.078, \mathrm{R}^{2} =0.83) \end{array} $$

and

(5) $$\begin{array}{*{20}l} g_{\text{fur}}(x) &= 1.545*exp\left(\left(\frac{x+0.1617}{1.761}\right)^{2}\right),\\ &\quad(\text{RMSD}=0.0095, \mathrm{R}^{2} =0.99) \end{array} $$

for the plaster cast and the fur-covered model, respectively. All fits had a single degree of freedom, the other goodness-of-fit statistics are stated in brackets behind the approximating functions. The Gaussian exponential approximation of the plaster cast’s HQIratio (Equation 4) show considerably inferior goodness-of-fit statistics compared to all other approximations. Its R ^2^ value of 0.83 and RMSD =0.078 face R ^2^ values of 0.99 and RMSD ≤0.0432. Both approximations of the fur-covered model’s HQIratios were suitable referring to the goodness-of-fit statistics, but the Gaussian exponential fit comes with three times smaller root-mean-square-deviation. The polynomial fit (dotted purple line in Figure 1) shows a local minimum between the original values belonging to 20 cm/s and 30 cm/s. In Figure 1 all approximations are displayed. Hereby in both models the inferior one is illustrated as dotted line.

### Pixelwise differences in standstill

For both cow models the fluctuation criteria SumDiff

(6) $$ \text{SumDiff}\, := \,\frac{1}{N_{0}-1}*\sum_{i=1}^{N_{0}-1} \left|\text{image}_{i+1}-\text{image}_{i}\right|  $$

and the pixel-wise calculated standard deviation pwStd (section ‘Material and methods’, ‘Comparison criteria and statistical methods’, *Pixelwise differences in standstill*) showed significant differences in medians between pixel belonging to “Interior” or “Boundary” (Table 2). The effect sizes for the criterion pwStd exceeded the effect sizes for SumDiff. According to (Cohen 1988), effect sizes in SumDiff were small (*η*^2^=0.013 for plaster cast and *η*^2^=0.017 for fur-covered model), while effect sizes for pwStd were medium (*η*^2^=0.111 for plaster cast and *η*^2^=0.096 for fur-covered model). The cow model significantly affected both criteria within both regions. The effect could be considered large within “Interior” (*η*^2^=0.232 with SumDiff, *η*^2^=0.235 with pwStd) but very small within “Boundary” (*η*^2^=0.006). Additionally, the fur color had a significant effect in both criteria (Table 3). Both criteria showed large effect sizes (*η*^2^=0.472).

**Table 2:** **Descriptive statistics of pixel-wise added differences (SumDiff) and pixel-wise standard deviation (pwStd) in depth values for plaster cast as well as fur-covered model recorded in standstill**

**Material**	**Plaster cast**			**Fur-covered model**		
**Criterion**	**N**	**SumDiff**	**pwStd**	**N**	**SumDiff**	**pwStd**
median “Interior”	10903	0.004	0.003	7920	0.006	0.006
median “Boundary”	505	0.006	0.029	425	0.013	0.017
max		0.678	0.673		0.688	0.678
“Interior”		0.678	0.072		0.579	0.670
“Boundary”		0.676	0.673		0.688	0.678
min		0	0.002		0.001	0.002
“Interior”		0.002	0.002		0.002	0.002
“Boundary”		0	0.003		0.001	0.004
mean		0.004	0.013		0.004	0.014
“Interior”		0.008	0.009		0.007	0.008
“Boundary”		0.049	0.099		0.097	0.141
		Effect sizes *η* ^2^ (*p*=0.02)				
Grouping Variable		SumDiff		pwStd		
region	Plaster Cast	0.013		0.111		
region	Fur-cov. M.	0.017		0.096		
model	“Interior”	0.232		0.235		
model	“Boundary”	0.006		0.006		

The area of both models had been disjointed in “Interior” and “Boundary”. The numbers of pixel belonging to each group are given in column 2 and 5. Since the data (SumDiff, pwStd) is skewed, the median is preferable as a measure of center. Nevertheless, the means are given for the sake of completeness. The differences in medians of SumDiff and pwStd are significant (p=0.02) both between regions “Interior” and “Boundary” for the two models and between models for the regions “Interior” and “Boundary”. Effect sizes *η* ^2^ are given in rows 13, 14 for the grouping after “Interior”/“Boundary” and in rows 15, 16 for the grouping after models, respectively.

**Table 3:** **Descriptive statistics of pixel-wise added depth value differences (SumDiff) and standard deviation (pwStd) for the fur-covered model recorded in standstill to compare between black and white fur**

**Criterion**	**N**	**SumDiff**	**pwStd**
median “Interior White”	6362	0.003	0.002
median “Interior Black”	1558	0.007	0.006
*η* ^2^ (*p*=0.001)		0.472	0.472
max		0.579	0.670
“White”		0.008	0.051
“Black”		0.579	0.670
min		0.002	0.002
“White”		0.002	0.002
“Black”		0.003	0.003
mean		0.007	0.008
“White”		0.003	0.003
“Black”		0.008	0.009

The numbers of pixel belonging to “Interior White” and “Interior Black” are given in column 2. Since the data is skewed, the median is preferable as a measure of center. The means are given for the sake of completeness. The differences in medians of SumDiff and pwStd between “Interior White” and “Interior Black” are significant (p=0.001). Effect sizes *η* ^2^ are given in row 4.

### Precision of coordinate determination

Table 4 shows the development of precision criterion RpV

(7) $$ { \small\begin{aligned} \text{RpV}_{\text{velocity}}\, := \,\frac{\max(\text{X-coordinates})- \min(\text{X-coordinates})}{N_{\text{velocity}}} \end{aligned}}  $$

**Table 4:** **Range per number of values calculated for X-coordinates at different velocities RpV** $\boldsymbol {{~}_{\text {velocity}} = \frac {max(coord{.})-min(coord{.})}{N_{\text {velocity}}}}$ **, where** ***N***_***velocity***_ **is the number of images with determined X-coordinates at the corresponding velocity (standstill,** ***10*** **cm/s,** ***20*** **cm/s,** ***30*** **cm/s)**

**Material**	**Body part**	**RpV**_**0**_	**RpV**_**10**_	**RpV**_**20**_	**RpV**_**30**_	**quadr.coeff.**	**RMSD**	**R** ^**2**^
	Isc.Tub., L	0.008	0.038	0.044	0.061	0	0.008	0.96
	Dish, L	0.014	0.062	0.072	0.1	-0.01	0.013	0.96
plaster	Tail	0.014	0.062	0.069	0.087	-0.01	0.012	0.95
cast	Dish, R	0.013	0.067	0.069	0.1	-0.01	0.018	0.92
	Isc.Tub.,R	0.018	0.039	0.046	0.065	0	0.006	0.97
	BB30	0.0011	0.0108	0.0156	0.026	0	0.001	0.98
	Isc.Tub.,L	0.002	0.071	0.3	2.5	0.53	0.405	0.96
fur-	Dish,L	0.001	0.106	0.286	5.0	1.15	0.998	0.94
covered	Tail	0.002	0.078	0.191	0.75	0.12	0.092	0.98
model	Dish,R	0	0.036	0.048	1.5	0.35	0.33	0.93
	Isc.Tub., R	0.001	0.029	0.095	2	0.47	0.40	0.94
	BB30	0.0005	0.0142	0.0476	0.25	0.05	0.002	0.57

The seventh column contains the quadratic coefficients of the polynomial approximation of the vectors (*RpV*_0_, RpV_10_, RpV_20_, RpV_30_) for all considered body parts (abbreviated in column 2). The medians of the quadratic coefficients differ significantly between plaster cast and fur-covered model (p=0.05, median_plaster_=-0.005, median_fur_=0.41). The last two columns contain the goodness-of-fit statistics root mean square deviation (RMSD) and coefficient of determination (R ^2^). All fits had a single degree of freedom.

with increasing velocity for every considered body part and both models (section ‘Material and methods’, ‘Comparison criteria and statistical methods’, *Precision of coordinate determination*). Additionally, the goodness-of-fit statistics of the polynomial approximations of the vectors (*RpV*_0_,RpV _10_, RpV _20_, RpV _30_) are given. Except from the fit to RpV values belonging to BB30 measured with the fur-covered model, the coefficients of determination range from R ^2^=0.92 to R ^2^=0.98. The RMSD varies between 0.001 and 0.018 for the plaster cast and between 0.092 and 0.998 for the fur-covered model, again except the point BB30. The goodness-of-fit statistics for BB30 measured with the fur-covered model are noticeable, as RMSD =0.002 and R ^2^=0.57 are significantly lower than the values within the fits belonging to the fur-covered model. Furthermore, the R ^2^ value is significantly lower than the corresponding values of all other approximations. The quadratic coefficients of the RpV-approximations belonging to the plaster cast were close to zero (median_plaster_= −0.005). For the fur-covered model they reached from 0.05 to 1.15 (median_fur_=0.41). The imprecision criterion RpV grew significantly (*p*=0.05) faster with increasing velocity when it came to the fur-covered model. The size of the model’s effect was very large (*η*^2^=0.846).

## Discussion

This study provided four possible measures to quantify the effects of fur in contrast to a homogeneous plaster surface, fur color, and velocity.

### Proportion of high quality images (HQIratio)

Both surface materials showed loss in image quality measured via HQIratio due to animal movement. This was to be expected, as TOF cameras are prone to motion artifacts. As explained in section ‘Material and methods’, ‘Time-Of-Flight Technology’, the depth values were calculated using four signals *S*_1_,…,*S*_4_. Motion artifacts occur when objects move significantly during the acquisition of *S*_1_,…,*S*_4_ (Hansard et al. 2012).

However, the behavior in decrease of image quality differed significantly between the models. In this study, no velocities greater than 30 cm/s were considered. For such speeds, no images of the fur-covered model would have passed the quality tests implemented in the software (see section ‘Material and methods’, ‘Software’). Even at 30 cm/s only four usable images remained for that model as can be seen from Table 1. It was not expected that usable images of the model at a cow’s assumed normal walking pace of about 111 cm/s (≈4 km/h) could be acquired. To quantify the differences, approximating functions for the vectors (HQIratio_0_,HQIratio_10_,HQIratio_20_,HQIratio_30_) were determined. As the velocity was constantly increased by 10 cm/s per step, a quadratic behavior of the acceleration was to be expected. Therefore, approximations of the form *α*∗*x*^2^+*β*∗*x*+*γ* were calculated. The HQIratio values related to the fur-covered model (Table 1, rightmost column) indicated a faster decrease. Therefore, the behavior of the HQIratio_velocitiy_ vectors was in addition approximated by a Gaussian exponential function $K*exp\left (-\frac {(x-L)}{M}\right)$, and goodness-of-fit statistics of the approximating functions were compared. The minimal value of the polynomial fit to the fur-covered model’s HQIratios (Equation 3) was lower than the value for 30 cm/s. The polynomial approximation’s goodness-of-fit statistics were, nevertheless, quite as suitable as the ones belonging to the exponential approximation (Equation 5). This fact and the position of the polynomial’s minimum between the third and last original value could be explained by the limited number of HQIratio values, that had been considered. The approximation model had no original value beyond 30 cm/s to predict the decay, but the course could be described very well within the smaller velocities. Looking at the plaster cast’s approximations, the polynomial (Equation 2) is superior to the exponential fit (Equation 4). This was mainly caused by the HQIratio belonging to 10 cm/s, as it is considerably smaller than the HQIratio in standstill, but nearly equal to the HQIratio calculated for 20 cm/s. On the one hand, an unobserved effect during the recording of the plaster cast at velocity 10 cm/s might have caused this outlier. On the other hand, the image quality might show a polynomial instead of an exponential decrease with increasing velocity due to the homogeneity of the plaster cast’s surface. The fact, that the model was moving at all, seemed more meaningful than the actual velocity. A considerable amount of high quality images could be gathered even at the highest considered speed. With the fur-covered model on the contrary, every step in acceleration caused a substantial additional loss in image quality. This indicated, that the velocity would have to be kept as low as possible, when moving cows are to be recorded with the SR4K.

To quantify the effect of the surface material in motion, coefficients of approximations of the same type had to be compared between models. The Gaussian exponential approximation for the plaster cast should not be used in this comparison, because its goodness-of-fit statistics were clearly inferior. Concerning the first acceleration steps, the degree 2 polynomials were good fits for both models. As the coefficient with the *x*^2^ term had the most impact on a polynomial’s growth, the much faster decrease caused by the fur-covered surface in contrast to the plaster surface could be quantified by the quotient of the quadratic coefficients of *P*_fur_ and *P*_plaster_. That gives $\frac {0.1532}{0.0368}\approx 4.16$.

### Pixelwise differences in standstill (SumDiff, pwStd)

SumDiff (Equation 6) and pwStd were measures for pixel-wise deviation in depth values. As only recordings in standstill had been used for the calculation, these comparison criteria were independent of velocity and allowed to analyze the differences between models, that were caused strictly by surface material.

Mixed phases are produced when infrared light with different phase shifts was observed by one pixel. As an implication, the depth values were calculated from an superposition of multiple reflected signals. Such multipath errors had been expected to be a problem at the cow models’ boundaries, as this error generally increased as the objects surface’s normal deviates from the optical axis of the camera (Hansard et al. 2012). Therefore, the cow area was split up in the regions “Boundary” and “Interior” to reach better comparability. The grouping after regions within models and the grouping after models within regions effected both criteria significantly. Especially the size of the model effect within “Interior” was large (*η*^2^=0.23). This could be interpreted as a quantification of the effect of fur surface on TOF depth measurement precision. Due to the structure of fur, an augmented refraction of light occurred and less accurate TOF depth measurement was to be expected. Pixelwise deviation increased at the edge of the cow model’s area. Within “Boundary” only a small model effect could be observed. A plausible explanation was, that for both models the depth measurement within “Boundary” was already less accurate due to mixed phases, and therefore the surface structure had hardly an impact, whereas the accurate depth measurement within “Interior” was strongly affected by the fur.

It had additionally been distinguished between black and white fur within “Interior” of the fur-covered model. The fur color also had a significant effect in both criteria, and the effect sizes were very large (*η*^2^=0.472), probably caused by different absorption coefficients of black and white fur. The absorption coefficient was the quotient of the electromagnetic radiation which a body absorbed and the electromagnetic radiation it was exposed to. It ranged between 0 and 1. The exact absorption coefficients for white and black fur were not determined in this study, but assuming a higher absorption coefficient for black fur was reasonable. For example a surface of the carbon black and white marble had absorption coefficients ≈0.96 and ≈0.46, respectively (Baehr and Stephan 2004). The infrared signal reflected from the black fur had lost more intensity when it returned to the sensor inside the TOF camera than the signal reflected from the white fur (MESA-Imaging 2013a). Therefore, the depth measurement varied in quality. As the fur needed to be glued to the model to avoid that measurements became unrepeatable due to displacements of the coat, only one coat was tested. The effect sizes might depend on the specific coat texture of this real cow fur. Then again, using different coats might have caused difficulties in distinguishing between the effects of the animal and the fur color.

As pwStd is based on quadratic differences in contrast to the absolute differences used to calculate SumDiff, in pwStd larger differences in depth value gain more weight than in SumDiff. That might explain the smaller differences in medians and the less strong effects on SumDiff than pwStd when it came to a comparison between the models or the regions.

Smoothing the images was not considered in this study, as the effects on the original recordings had been of interest. Specific smoothing could be a possibility in an image processing application to handle the differences between black and white fur, at the risk of losing information about the surface shape.

### Precision of coordinate determination (RpV)

RpV (Equation 7) was a measure of imprecision concerning the determination of X-coordinates. The original software applied to cows in an electronic feeding dispenser had shown 1.5% error rate (Salau et al. 2014) in the detection of ischeal tuberosities, dishes of the rump, and tail. RpV had been calculated for all velocities with regard to the automatically determined X-coordinates of these five body parts and additionally for BB30. In both cases RpV rose while the models accelerated. However, for the plaster cast only a linear growth could be observed, as the quadratic coefficients were all close to zero, whereas RpV increased quadratically for the fur-covered model. Therefore, the fur affected the loss of precision due to velocity very strongly (*η*^2^=0.846). Indeed, the imprecision in coordinate determination in standstill was higher for the plaster cast, than the fur-covered model. But differences in the geometrical shape of the models could not have been excluded as reasons for more erroneous coordinate determination concerning the plaster cast. A noticeable fact was, that BB30 showed not only the smallest RpV values for all velocities in both models, but also a large difference in quadratic coefficients compared to the other body parts when it came to the fur-covered model. This indicated, that this body part could be determined most accurately and exhibited the least loss of precision due to velocity. A reason for this might be, that of all considered body parts only BB30 lay in “Interior” instead of “Boundary”, where the TOF depth measurement was more reliable. It has to be taken into account, that the coefficient of determination for the approximation with a quadratic polynomial corresponding to BB30 on the fur-covered model was inferior compared to the approximations belonging to the other body parts. It could be questioned, if the calculated quadratic coefficient was meaningful.

### Discussing TOF usage

In dairy production fur surfaces had to be considered and no influence on the fur color could be taken. It had to be analyzed how the application of TOF technology could lead to dependable results. Dairy cows’ BFT was successfully estimated from TOF recordings in (Weber et al. 2014) with the limitation, that cows were only recorded in standstill. Furthermore, traits were only extracted from one dimensional sections through the recorded cow surfaces and not from two dimensional areas on the surfaces. The reason was, that differences in depth measurement between black and white fur could be corrected in a more controlled way when only one dimension was considered. Principal descriptors for i.e. body condition scoring were located in the tail head area (Ferguson et al. 1994) where the effects of fur and velocity turned out to be strong. Thus, it had to be expected, that assessing BCS or BFT from TOF recordings of moving animals would be erroneous. The restriction of analyzing traits from “Boundary” only from recordings collected during feeding or milking is a serious limitation for a monitoring system. However, the effects of fur and velocity were noticeably smaller in “Interior”, hence the TOF camera might be applicable for the determination of the backbone in moving cows and lameness detection via back posture analysis as in (Hertem et al. 2014). Yet, it was questionable if a TOF camera could be a superior choice for dairy farming application, as the Kinect was cheaper, did not show differences in depth measurement between black and white fur, and produced little motion artifacts. With regard to the latter should be mentioned, that real-time preprocessing methods to compensate motion artifacts in TOF recordings have been introduced (Hoegg et al. 2013). Considering the effect of fur again, Kinect’s and SR4K’s performance on measuring stuffed animals, small fur-covered animal models, and other test objects had been examined in (Hansard et al. 2012). It has to be mentioned, that synthetic fur’s structure differs from that of real fur. But with both fur-covered test objects the RMSD of depth accuracies between Kinect and SR4K were comparable. The Kinect’s performance over all test objects turned out to be worse than that of SR4K.

The next generation of the Kinect is a TOF camera, but it is equipped with a novel image sensor (Lau 2013). Every pixel is divided in half, the pixel halves are ready to absorb reflected light alternately, and the absorbing time of the first half is aligned with the pulsing of the laser. During the time the first half is rejecting incoming light, the second half is absorbing, and the laser is off. Consequently, the distribution of received photons among both pixel halves changes with the distance between camera and object and is used to calculate depth values. A renunciation of the control signals *S*_1_,…,*S*_4_ could limit motion artifacts, because there are less possibilities for the object to move during calculation. If proportions of light were absorbed by the object’s surface and did not return to the sensor, both pixel halves were affected equally, and the distribution was not altered. This would reduce black-white-differences in depth measurement significantly, as they were a consequence of the absorbing coefficients of black, respectively, white fur. The next Kinect has not been available while data collection for this study was carried out, but it promises to be an affordable alternative to both Kinect and the current generation of TOF depth sensors.

## Conclusion

This study introduced criteria to quantify the effects of fur and animal movement. The experimental indoor test scenario included two cow models with fur and plaster surface, respectively. According to the criteria concerning pixel-wise deviation (SumDiff, pwStd), the effect of the fur surface in contrast to a more homogeneous surface on TOF measurement was large. Additionally, crucial differences related to fur color were observed, as criteria medians were two to three times higher with black than white fur. In any application of TOF cameras, the velocity of the recorded animals needed to be controlled, because in the analysis of moving models, the impact of the fur surface became even more decisive: With increasing velocity the proportion of high quality images (HQIratio) dropped four times faster due to fur, and furthermore, the fur caused quadratic loss of precision in coordinate determination (RpV) in contrast to a linear behavior without fur. The latter was a problem especially at the edge of the cow model’s area, i.e. the tail head region. However, coordinate determination was sound in the middle of the cow’s back and hardly affected by velocity or fur. It was shortly discussed, if TOF depth sensors could compete with the Microsoft Kinect 3D camera when it comes to studies dealing with traits from the cow area’s interior. At this, an outlook on the next Kinect camera generation was given. Its new type of TOF sensor seemed to be a noticeable improvement to both current TOF sensors and Kinect.

## Material and methods

### Time-Of-Flight Technology

The SR4K (Mesa Imaging AG) emits infrared light (modulated signal frequency *f* = 30 MHz), which is reflected by the object. From four phase control signals *S*_1_,…,*S*_4_ with 90 degree phase delays from each other the collection of electrons from the detected reflected infrared signal is determined. Let *Q*_1_,…,*Q*_4_ represent the amount of electric charge for *S*_1_,…,*S*_4_, respectively. Using the four phase algorithm, the phase difference *t*_*d*_ is estimated as $t_{d} = \text {arctan}\frac {Q_{3} - Q_{4}}{Q_{1} - Q_{2}}$. The distance *d* between object and camera is calculated from the phase shift with the following formula: $d = \frac {c}{2 f}\frac {t_{d}}{2\pi }$, whereas *c* and *f* denote the speed of light and the signal frequency, respectively (Hansard et al. 2012). The camera’s range is 0.8 to 5 m (MESA-Imaging 2013a). Its accuracy of measurement over this calibrated range is 1 cm (for the 11×11 central pixel). It is recording up to 54 images per second with a resolution of 176×144 pixel depending on the exposure time and has 43.6^o^ horizontal and 34.6^o^ vertical field of view. SR4K provides distance and (*x*,*y*,*z*) coordinate data, amplitudes, confidence maps as an estimate of reliability, and SwissRanger streams (srs) consisting of sequences of images as output according to user’s choice. The camera was used with default settings.

### Recorded cow models

Two cow models were recorded with a SR4K TOF camera in September 2012 at the Institute for Agricultural Engineering and Animal Husbandry of Bavarian State Research Center for Agriculture (BSRCfA) in Grub (Germany). Recording (details in ‘Installation and recording’) of both models were taken from top view in standstill and motion.

**Figure 2:**
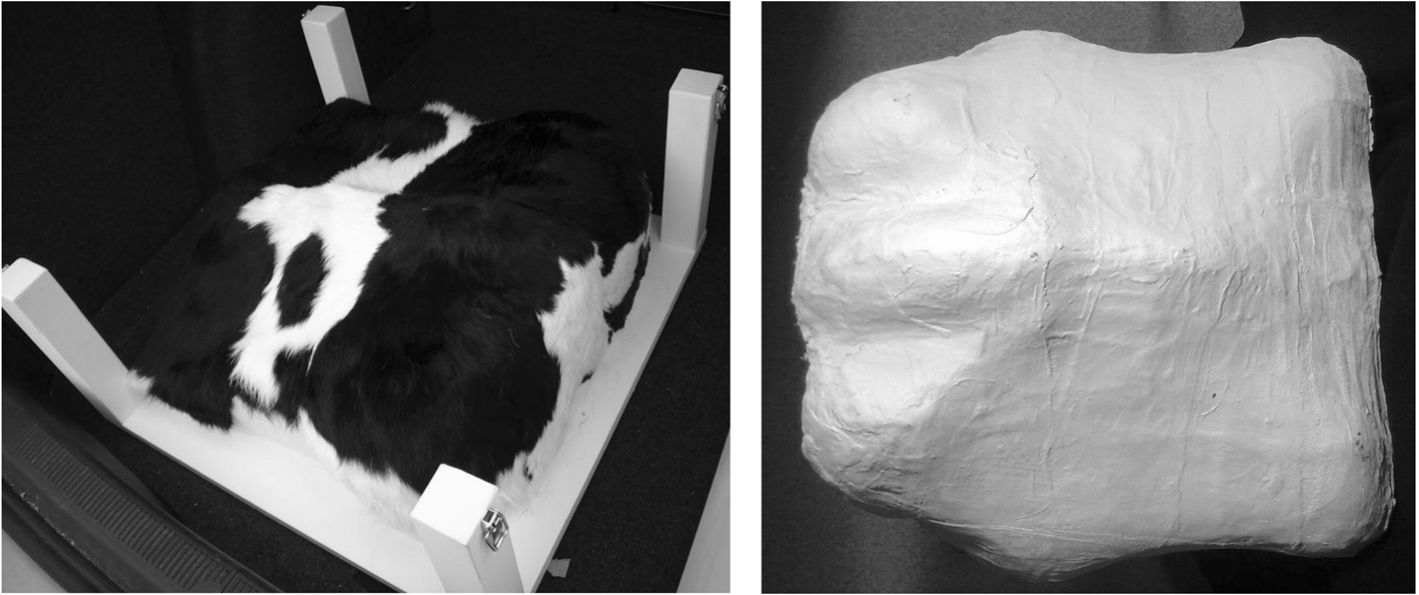
**The two recorded cow models.** Left: Fur-covered Model; Right: Plaster Cast.

A model of a cow’s lower back was build at BSRCfA from solid, synthetic material using CNC (computer numerical control) carving (width: 0.5 m, length: 0.5 m, height: 0.15-0.22 m). It had a tail, ischeal tuberosities and a lower backbone but no hipbones and was not modeled after a real cow (Figure 2, left). A black and white real Holstein-Friesian’s fur was permanently glued to the model. The fur-covered model was firmly mounted on a board (0.6×0.6 m ^2^) with wooden beams (0.05×0.05 m ^2^, height: 0.25 m) at the corners. Since the fur could not be removed from the model without destroying it, no other furs were used for testing. Originally, this model was intended for another purpose within a study related to body condition determination (Weber et al. 2014; Salau et al. 2014). Hipbones were not included in the model, because they were irrelevant then. The model nevertheless shows most of the points of interest (ischeal tuberosities, dishes of the rump, tail, backbone) that could be determined by the software described in (Salau et al. 2014). Therefore, the model was reused in this context.

Later, since no fur-less version of the model was available, a plaster cast was taken from a Holstein-Friesian cow’s lower back to obtain a portable model of a real cow’s shape as a negative control for fur (Figure 2, right). This was done at the research farm Karkendamm of Christian-Albrecht-University (CAU) in Kiel (Germany). The lower back was greased with petrolatum to protect fur and skin of the animal. Afterwards this area was uniformly covered with several layers of wet plaster bandages. The covered area included base of the tail, ischeal tuberosities, lower backbone and lower back. The hipbones were not included. The bandages reached approximately 15 cm down the animal’s side. A blow-dryer was used to fasten the drying, before the plaster cast (length: 0.56 m, width: 0.55 m, height: 0.16-0.22 m) was lifted of the cow.

### Installation and recording

**Figure 3:**
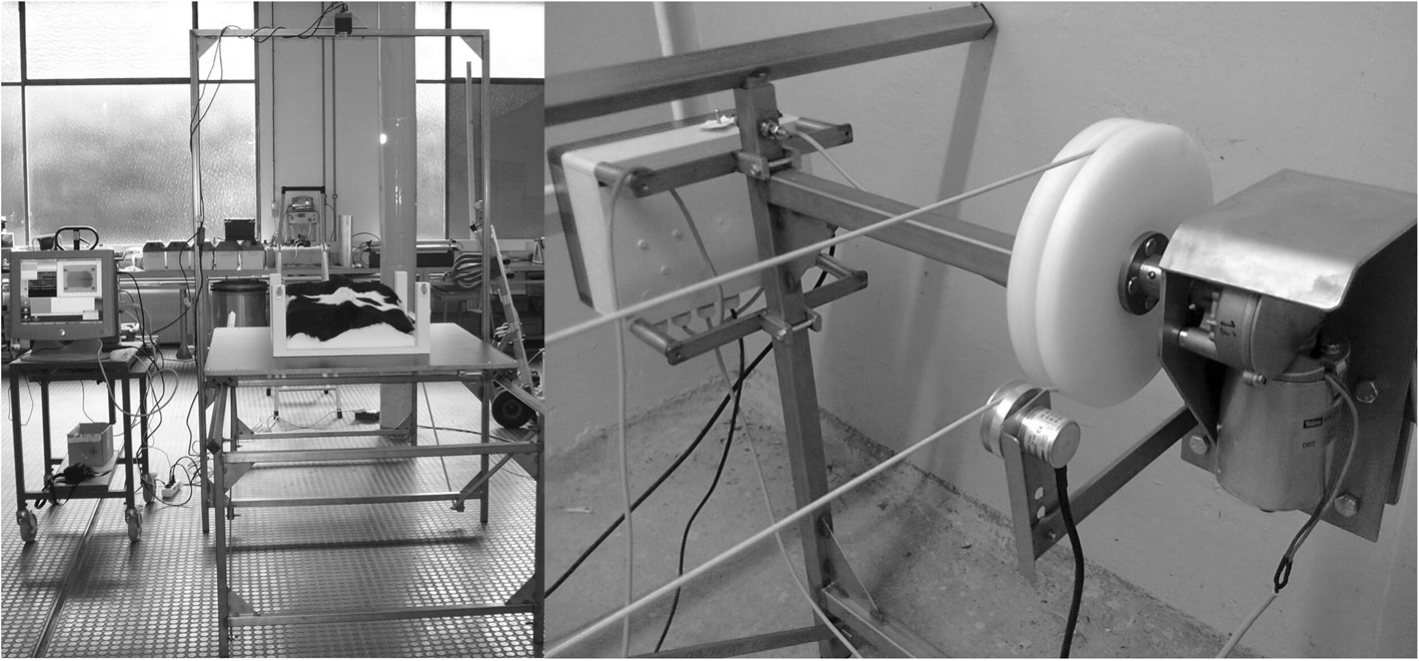
**Installation for recording in controlled velocities.** Left: Framework with fur-covered model on a wooden plate placed on running rails. SR4K mounted in top view; Right: Motor (background), impeller wheel and rope to tow the wooden plate.

A metallic frame with two horizontal running rails was build by BSRCfA (length: 3 m, width: 1.04 m, height: 0.8 m). A wooden plate (1.14×1.14 m ^2^) was placed on the running rails which could be towed by a rope stretched up to an impeller wheel. The motor could be regulated with a control panel that also displayed the velocity. At the end of the running rails the plate was stopped automatically, and the moving direction could be changed by a switch. The construction was supported by a vertical frame (width: 1.27 m, height: 2.13 m) above which’s center line the TOF camera was attached in top view (Figure 3).

Recording took place indoors at BSRCfA to exclude the influences of direct sunlight or insects. In Grub a 2-core system having 3.43 gb RAM with the recording software (see section ‘Software’) developed at the Institute of Animal Breeding and Husbandry of CAU was used for recording. SwissRanger streams of both models were recorded in standstill and at the controlled velocities 10 cm/s, 20 cm/s and 30 cm/s. The camera stayed in fixed position with 1.28 m distance between sensor and the wooden plate. Additionally streams of the wooden plate without any model on it were recorded to capture the completely empty scenery.

### Software

#### Originally developed software

At CAU software was developed to record cows in an electronic feeding dispenser and automatically extract body traits (Salau et al. 2014). The software firstly calculated scenery information out of a number of images of the completely empty scenery. It then could decide automatically if an image showed a cow’s lower back. These images were segmented and stored for further processing, all others were deleted. Subsequently, the body parts ischeal tuberosities, base of the tail, dishes of the rump, hipbones, and backbone were determined automatically. The software tested the segmentation results and the coordinates of body parts directly after their calculation (for details see (Salau et al. 2014)). Images failing any test were deleted.

#### Necessary software modifications made in this study

The streams recorded from the cow models were used as virtual camera and analyzed with this software. As the models differed from real cows, some slight modifications to the software were necessary:

1. The basis for the automated decision that the image showed a cow’s lower back had been, that the area covered with the cow’s body exceeded the lower image border. As the models did not reach the image border, an additional rectangle (Figure 4, middle) had been temporarily added to every image to close the gap and to avoid, that all images were deleted. After successful segmentation the rectangle was removed again (Figure 4, right).

**Figure 4:**
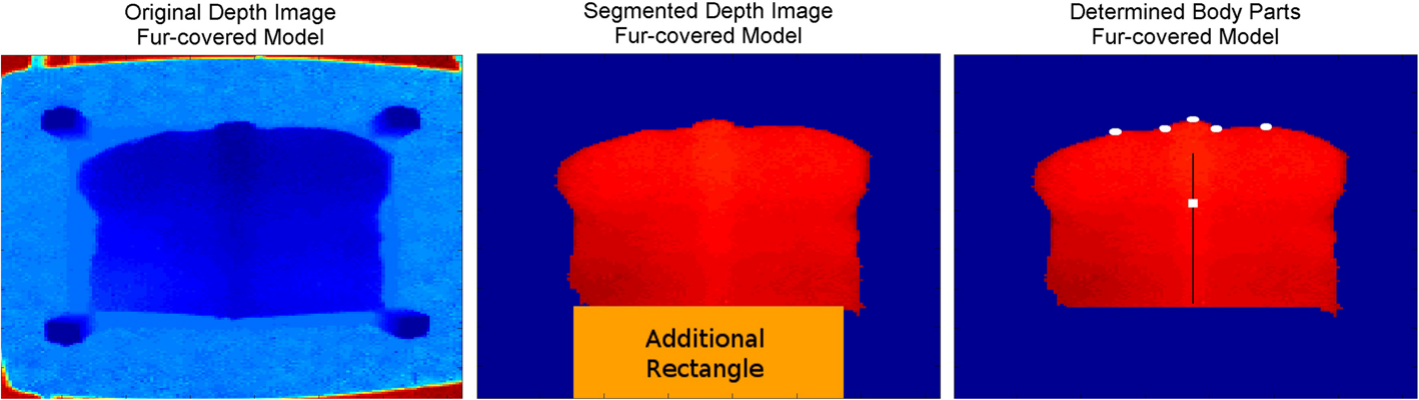
**All three illustrations were prepared using the MATLAB function imagesc and its default color scale.** Left: Original depth image, showing the fur-covered model on the wooden plate. It was mounted on a board. Middle and Right: The subsequent image processing steps. Middle: A rectangle has been added to the depth image. This modification of the original software was necessary to prevent the image from being deleted, because the models in contrast to real cows did not reach the image’s lower edge. Afterwards the automated segmentation has set all background to zero (blue). Right: The backbone (black line), ischeal tuberosities, dishes of the rump and tail (white dots) and BB30 (point on the backbone in 30 pixel radius from the tail, white rectangle) have been determined automatically.
2. The cow was separated from the background by subtracting the averaged empty scenery and using the height differences between the cow and the floor. Both models reached a maximum height of 0.22 m above the wooden plate (This served as the floor in the present scenario.), thus the tolerances had to be adapted. In case of the fur-covered model the position of the wooden beams (section ‘Material and methods’, ‘Recorded cow models’, Figure 4, left) relative to the model had once to be specified manually and their removal had to be added to the automatic segmentation.
3. As hipbones were not included in both models, their automatic determination had to be removed from the software. Instead, the point on the backbone in 30 pixel radius from the tail (BB30) was determined, in order to include a measurement point in the comparison, that was not positioned at the edge of the cow area (Figure 4, right).

### Comparison criteria and statistical methods

MATLAB presents the images as matrices with 176 rows and 144 columns. Counting rows and columns starts at the left upper corner. The vertical midline runs between columns 72 and 73. The algorithms of the software developed in (Salau et al. 2014) work the images row-wise and column-wise from the left upper corner. To exclude this running direction as reason for left-right-differences in the extracted comparison criteria, the analysis had been repeated with all images mirrored on the vertical line between column 72 and 73.

#### Proportion of high quality images

As the camera stayed in a fixed position during recording the number of images showing the cow model decreased with increasing velocity (Table 1). These numbers will be called *C*_0_ belonging to the recording in standstill and *C*_10_, *C*_20_, and *C*_30_ belonging to the recordings at 10 cm/s, 20 cm/s, and 30 cm/s, respectively. Both models were recorded for four minutes in standstill. For all velocities both cows model were recorded passing the camera five times. As explained in section ‘Software’, *Originally Developed Software* various quality tests had been integrated in the software and all images failing any of these tests were deleted. The numbers of output images after applying the quality tests will be called *N*_0_,*N*_10_,*N*_20_, and *N*_30_. The quotient

$$\text{HQIratio}_{\text{velocitiy}}=\frac{N_{\text{velocitiy}}}{C_{\text{velocitiy}}} $$

(compare Equation 1) is the ratio of High Quality Images in relation to recorded images. For both models the behavior of HQIratio_velocitiy_ with increasing velocity was analyzed by approximating the vector (HQIratio_0_,HQIratio_10_,HQIratio_20_,HQIratio_30_) with a quadratic polynomial *α*∗*x*^2^+*β*∗*x*+*γ* on the one hand and with a Gaussian exponential function $K*exp\left (-\frac {(x-L)}{M}\right)$ on the other hand. For every approximation the root-mean-square-deviation RMSD, coefficient of determination R ^2^, and degrees of freedom were calculated as goodness-of-fit statistics. The RMSD in general is the sample standard deviation between the actually observed values *y*_*t*_ and the values $\widehat {y}_{t}$ calculated by the approximation $\text {RMSD}=\sqrt {\frac {1}{n}*\sum _{t=1}^{n} \left (y_{t}-\widehat {y}_{t}\right)^{2}}$, where *n* denotes the sample size. For all approximations the MATLAB Curve Fitting Toolbox (The MathWorks Inc 2014a) was used.

#### Pixelwise differences in standstill

Considering only the images in standstill the pixel-wise deviation in depth values was analyzed for both models. For this purpose the pixel-wise absolute differences of every two consecutive images were taken, summed up, and divided by the number of summands (*N*_0_ as defined in section ‘Material and methods’, ‘Comparison criteria and statistical methods’, *Proportion of high quality images*):

$$\text{SumDiff}\, := \,\frac{1}{N_{0}-1}*\sum_{i=1}^{N_{0}-1} \left|\text{image}_{i+1}-\text{image}_{i}\right|, $$

(compare Equation 6). This resulted in a matrix containing the values of the criterion SumDiff for every pixel. Additionally for every pixel the standard deviation (pwStd) in depth values was calculated. While pwStd used the quadratic aberration around the mean depth value, in SumDiff the variation from image to image was taken neglecting the pixel-wise depth values’ mean.

**Figure 5:**
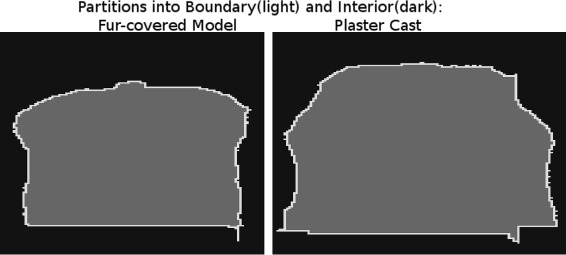
**“Interior” and “Boundary”.** All pixel with a neighborhood of radius 1 that intersected with the background (black) and the cow-area (gray) belonged to “Boundary”. All other pixel of the cow area were defined to be “Interior”. Left: Fur-covered Model; Right: Plaster Cast.

**Figure 6:**
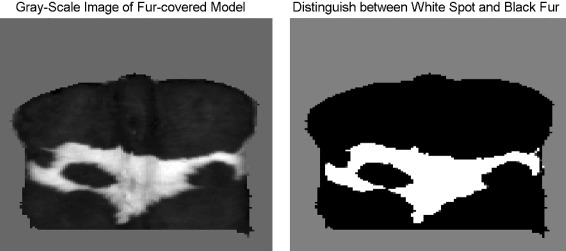
**Distinction between black or white fur.** Left: Segmented gray scale image of the fur-covered model; Right: The white spot is defined as all pixel with gray scale ≥25.

Each image could be split up in foreground (covered by cow model) and background (set to zero). Abrupt changes in the distance between recorded object and camera led to more possible ways for the infrared light to be reflected and return to the sensor and to less accurate depth measurement. The problem of erratic depth values along steep edges is a well known problem of TOF-cameras; compare (Langmann et al. 2012). The pixel-wise deviation in depth value was thus expected to be larger for pixel at the edge of the cow area. Visual inspection of depth maps revealed, that the main reflections or peaks in depth measurement occurred in an only one to two pixel wide area between background and cow area. Therefore, the foreground was split up in the disjoint areas “Boundary” and “Interior” (Figure 5). If a pixel’s neighborhood of radius one intersected with both the foreground and background, the pixel was considered “Boundary” (425 pixel fur-covered model, 505 pixel plaster cast). If the neighborhood was fully included in the foreground, the pixel was considered “Interior” (7920 pixel fur-covered model, 10903 pixel plaster cast). All images where tested once using this definition of “Boundary”. The effect of different boundaries was not tested. In the analysis of the fur-covered model, “Interior” was additionally partitioned in the disjoint areas “Interior Black” (6362 pixel) and “Interior White” (1558 pixel). A gray scale image of the amplitudes’ map was used to distinguish between black or white fur (Figure 6). All pixel with a gray scale value ≥25 were considered to belong to the white spot.

The Wilcoxon rank-sum test is a nonparametric version of the classical t-test. It compares the medians of the sample groups by examining the ranks of the data’s scores within both groups’ observations. The values of SumDiff and pwStd were considerably skewed and thus not normally distributed. Additionally, the grouping in “Boundary” or “Interior” (likewise “Interior Black” and “Interior White”) naturally led to unequal group sizes. Therefore, for both models Wilcoxon rank-sum tests instead of t-tests were performed to examine if the pixel’s position in “Boundary” or “Interior” had significant effect. Furthermore, all SumDiff and pwStd data collected from the regions “Interior” and “Boundary” was grouped after models, respectively, and Wilcoxon rank-sum tests were performed to look into the effect of the cow model on pixel-wise deviation. Concerning the fur-covered model additional Wilcoxon rank-sum tests (level of significance p =0.02) were performed to analyze the effect of the fur color. All group medians were calculated. In case significance was given, the ranked data was used to calculate the effect size $\eta ^{2} = \frac {SS\,due\,to\,grouping\,variable}{total\,sum\,of\,squares\,(SS)}$, which is the proportion of variance in the data explained by the grouping. For all statistical calculations the MATLAB Statistic Toolbox (The MathWorks 2014b) was used.

#### Precision of coordinate determination

The software automatically detected six points: ischeal tuberosities, dishes of the rump, tail, and the point on the backbone in 30 pixel radius from the tail (BB30, Figure 4, right). It was analyzed how strongly velocity effects the precision of coordinate determination. The X-coordinates were detected automatically in standstill and motion. Within each velocity their deviation was measured using the criterion RpV defined in Equation 7, and those RpV-values were compared between velocities. Due to the deviation in depth values the X-coordinates of body parts naturally were subject to 1 to 2 pixel fluctuation. This was analyzed with the criteria SumDiff and pwStd. As in this analysis solely fluctuation due to errors in the automatic determination of body parts was to be considered, in this comparison not the standard deviation in X-coordinates was used as criterion. Instead, the quotient of the X-coordinates’ range divided by the number of values (RpV: Range per number of Values) was taken as a measure of imprecision:

$${\small\begin{aligned} \text{RpV}_{\text{velocity}}\, := \,\frac{\max(\text{X-coordinates})-\min(\text{X-coordinates})}{N_{\text{velocity}}} \end{aligned}} $$

(compare Equation 7) with velocities 0,10,20,30 cm/s. Therefore, for each of the six considered body parts a vector (RpV _0_, RpV _10_, RpV _20_, RpV _30_) was calculated using the X-coordinates extracted from both models, respectively. These vectors were approximated with quadratic polynomials *α*∗*x*^2^+*β*∗*x*+*γ* using the MATLAB Curve Fitting Toolbox (The MathWorks Inc 2014a) and root-mean-square-deviations RMSD, degrees of freedom, and coefficients of determination R ^2^ were calculated as goodness-of-fit statistics. The quadratic coefficients *α* describe the polynomials’ growth behavior. To examine the cow model’s effect on the growth of imprecision a Wilcoxon rank-sum test was performed on the quadratic coefficients. The medians belonging to each cow model were calculated, and the effect size *η*^2^ was determined.

Except from the recordings in standstill, the models were moving vertically through the camera’s field of view. This implies, that Y-coordinates changed from image to image. This led to several sources of imprecision concerning the analysis of Y-coordinates. Therefore, it is excluded from the main article and presented in the Additional file 1.

### Declaration of adherence to ethical guidelines

The authors declare that the plaster cast was taken strictly following international animal welfare guidelines. The institutions the authors are affiliated with do not have research ethics committees or review boards. The cast was taken in a completely noninvasive manner. The cow was not forced into an unnatural body posture and was fastened for no longer than one hour. Feed was provided during the procedure. No corrosive, burning, unpleasant, extremely hot or cold substances were used.

## Additional file

Additional file 1 **Precision of automatically determined Y-coordinates at different velocities.** Supplementary Material to “Quantification of the effects of fur, fur color, and velocity on Time-Of-Flight technology in dairy production”, provided as pdf.

## Abbreviations

TOF: Time-Of-Flight, a principle of depth measurement (see (Hansard et al. 2012))
*η*^2^: Measure for the size of the effect of grouping data after a certain criterion, calculated from analysis of variance’s
SS: $\eta ^{2} = \frac {\mathrm {SS\,\,due\,\,to\,\,grouping}}{\mathrm {total\,\,SS}}$
*SS*: Sum of squares, $SS=\sum _{t=1}^{n} \left (y_{t}-\widehat {y}_{t}\right)^{2}$, *y*_*t*_ observed values, $\widehat {y}_{t}$ estimated values, *n* sample size
BCS: body condition score
BFT: Back fat thickness
SR4K: Swiss Ranger 4000, TOF camera produced by Mesa Imaging AG (MESA-Imaging 2013b)
HQIratio: The quotient of the number of images that passed the quality test by the number of images showing the cow model: HQIratio$_{\text {velocity}}=\frac {N_{\text {velocity}}}{C_{\text {velocity}}}$
P _plaster_, P _fur_, g _plaster_, g _fur_: Approximating polynomials and Gaussian exponential functions for HQIratio (Equations 2, 3, 4, and 5)
RMSD: Root-mean-square-deviation, $\text {RMSD}=\sqrt {\frac {1}{n}*\sum _{t=1}^{n} \left (y_{t}-\widehat {y}_{t}\right)^{2}}$, *y*_*t*_ observed values, $\widehat {y}_{t}$ estimated values, *n* sample size
R ^2^: Coefficient of determination
SumDiff: Sum of pixel-wise absolute differences of every two consecutive images, divided by the number of summands (Equation 6)
pwStd: pixel-wise standard deviation
RpV: Quotient of the X-coordinates’ range divided by the number of values (Equation 7)
BB30: Point on the cow models’
backbones in a radius of 30 pixel from the tail; *t*_*d*_: Phase delay
*S*_1_,…,*S*_4_: Phase control infrared signals emitted by SR4K to estimate phase delay *t*_*d*_
MHz: Megahertz
*Q*_1_,…,*Q*_4_: Amount of electrical charge for *S*_1_,…
,*S*_4_, respectively; *d*: Distance between object and TOF camera
*c*: Speed of light
*f*: Modulated signal frequency used by SR4K
srs: swiss ranger stream, data format generated by SR4K
BSRCfA: Bavarian State Research Center for Agriculture in Grub (Bayerische Landesanstalt für Landwirtschaft 2015) (Germany)
CNC: Computerized numerical control
CAU: Christian-Albrechts-University Kiel (Christian-Albrechts-Universität zu Kiel 2015) (Germany)
C _0_, C _10_, C _20_, C _30_: Number of images showing the cow model recorded at 0,10,20,30 cm/s
N _0_, N _10_, N _20_, N _30_: Numbers of images recorded at 0,10,20,30 cm/s that passed the quality tests
*α*∗*x*^2^+*β*∗*x*+*γ*: (approximating) quadratic polynomial
$K*exp\left (-\frac {(x-L)}{M}\right)$: (approximating) Gaussian exponential function
