# Supplementary material for: Quantification of the effects of fur, fur color, and velocity on Time-Of-Flight technology in dairy production
Source: Springerplus. 2015 Mar 26;4:144. doi: 10.1186/s40064-015-0903-0 (PMC4387134; doi:10.1186/s40064-015-0903-0)
Supplement: Supplementary file 1 — Precision of automatically determined Y-coordinates at different velocities. Supplementary Material to “Quantification of the effects of fur, fur color, and velocity on Time-Of-Flight technology in dairy production”, provided as pdf. [file 40064_2015_903_MOESM1_ESM.pdf]

RESEARCH

Precision of automatically determined Y-coordinates at different velocities

Supplementary Material to
"Quantification of the effects of fur, fur color, and velocity on Time-Of-Flight technology in dairy production"

Jennifer Salau[1*], Ulrike Bauer[2], Jan H. Haas[1], Georg Thaller[1], Jan Harms[2] and Wolfgang Junge[1]

[*]Correspondence: jsalau@tierzucht.uni-kiel.de
[1]Institute of Animal Breeding & Husbandry of Christian - Albrechts - University, Olshausenstraße 40, 24098 Kiel, Germany
Full list of author information is available at the end of the article

1 Background

1.1 Connection to main paper

The main paper examines the effects of fur, fur color, and velocity on Time-Of-Flight recordings. For this purpose a plaster cast of a cow's lower back and a fur-covered cow model were recorded in standstill and at 10, 20, and 30 cm/s. The study used a modified version of a software implemented to determine dairy cows' body condition ([1], [2]). This software automatically determined the models' ischeal tuberosities, dishes of the rump, and tail. These will in the following be referred to points of interest (POI). Several quality tests were implemented, and all images failing any test were deleted. The X- and Y-coordinates of the POI were automatically determined for high quality images only. The numbers of recorded images and high quality images were presented and analyzed in the main paper (Table 1 and Figure 1). Additionally, it was analyzed in detail, how the precision for the determination of X-coordinates for the POI varies with the velocity (Table 4).

1.2 Problems in the analysis of Y-coordinates

An analysis of the Y-coordinates was not presented in the main paper, because it was subject to several sources of imprecision. As the models' motion direction was parallel to the Y-axis, the Y-coordinates naturally changed from image to image except in the recordings in standstill. Therefore, it was not meaningful to measure the deviation of the automatically determined Y-coordinates within velocities as it was done with the X-coordinates. Coordinate determination was only performed on high quality images, and the quality tests integrated in the software had led to deletion of single images, thereby leaving gaps between high quality images. Although constant velocities were chosen, constant changes in Y-coordinates could in consequence not be expected.

Manually labeling the POI to gather "true Y-coordinates" was unfeasible due to time and manpower constraints, if a statistically sound number of images were to be tested. In addition, possible subjective influences caused by observers can not be excluded or controlled. Instead, "true Y-coordinates" could be calculated automatically, based on the POI starting position in each run, the time interval between images, and the given velocity. This method, however, was susceptible to systematical errors:

As the velocities were given in centimeters per second (cm/s), the distances were given in centimeters when time intervals in seconds were used for calculation. They needed to be transferred into pixel to be compared to the automatically determined Y-coordinates. The ratio between cm in the field of view (FOV) and pixel in the image varies with the distance from the camera. As the models' surfaces are not flat, the POI's exact distances from the camera were difficult to determine. Additionally, the camera's FOV naturally has the shape of a cone ([3]). In presenting the FOV as a rectangle, distortion increases towards it's edges. Pixel on the same plane perpendicular to the camera's line of sight (i.e. equal distance from the camera) vary in their widths and height in centimeters when one pixel is located in the center and the other at the edge of the image. This distortion effect could not be corrected afterwards, because the camera's internal projection on the rectangular image is unknown. As only integer numbers of pixel could be used as "true Y-coordinates", roundoff errors were also likely to occur.

2 Calculation of "true Y-coordinates"

The Y-coordinate of the point on the backbone 30 pixel away from the tail (BB30, see main paper section **4 Material and Methods**, *Software*) has per definition the Y-coordinate of the tail augmented by 30. It was hence not analyzed, and only the ischeal tuberosities, dishes of the rump, and the tail were taken into account.

The software described in [1] wrote the image acquisition time (in milliseconds) in every image's filename. The timestamps of all images for which coordinates for the POI could be calculated were extracted. The time differences Δt in seconds between them were determined. As the models were recorded while passing the camera five times, the respective first image of every run to show the POI was used as reference, and the Y-coordinates determined from it were used as start values. For all following images of each run, the distances Δd_{cm} that the POI should theoretically have moved away from the reference points during the corresponding Δt were calculated using

$$\Delta d_{\text{cm}} = \Delta t * \text{velocity}\,, \quad \text{velocity} \in \{0\,\text{cm/s}, 10\,\text{cm/s}, 20\,\text{cm/s}, 30\,\text{cm/s}\}. \tag{1}$$

The used Time-Of-Flight camera (Swiss Ranger 4000 by Mesa Imaging, [3]) has 34° opening angle in Y-direction. The camera was mounted 128 cm above the wooden plate on which the models were placed. For all POI the approximate height h over this wooden plate was determined. The difference $128 - h$ cm was used as distance between camera and the respective point of interest (presented in Supplementary Table 1).

To calculate the pixel's width in Y-direction, the rectangular triangle between the camera's line of sight (i.e. distance from the camera), the width in Y-direction $\text{Ywidth}_{\text{scenery}}$ of the whole scenery, and the boundary of the FOV's cone was used (Supplementary Figure 1): The tangens of half of the opening angle (17°) is the half of the scenery's width in Y-direction divided by the distance from the camera $128 - h$ cm. The scenery's width in Y-direction calculates to

$$\text{Ywidth}_{\text{scenery}} = 2 * \tan(17^{\text{o}}) * (128 - h)\,\text{cm}. \tag{2}$$

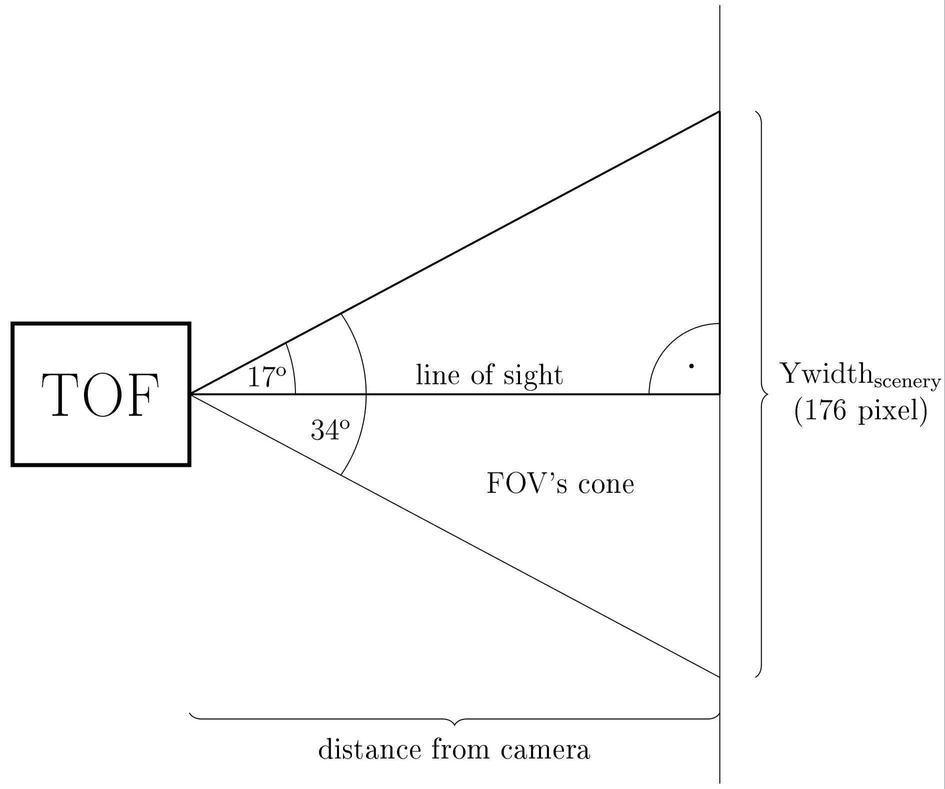

Supplementary Figure 1 Schematic representation: The TOF-camera's line of sight, the boundary of the cone of the field of view (FOV), and the scenery's plane of projection form a rectangular triangle. The tangens of the camera's half opening angle is equal to the ratio of the projection plane's distance from the camera and its width in Y-direction ($\mathrm{Ywidth}_{\mathrm{scenery}}$).

This number has to be divided by the number of pixel in Y-direction (176), to get a pixel's width in Y-direction, depending on the scenery's distance from the camera $128 - h$ cm:

$$\mathrm{Ywidth}_{\mathrm{pixel}} = \frac{1}{88} * \tan(17^{\mathrm{o}}) * (128 - h)\,\mathrm{cm}. \quad (3)$$

The differences in distance in centimeters Δd_{cm} have been calculated with Equation 1, but differences in pixel were needed:

$$\Delta d_{\mathrm{pixel}} = \frac{1}{\mathrm{Ywidth}_{\mathrm{pixel}}} * \Delta d_{\mathrm{cm}}. \quad (4)$$

To get the "true Y-coordinates" the distance differences in pixel $\Delta d_{\mathrm{pixel}}$ were rounded to integer values and added to the corresponding starting values. The difference between "true Y-coordinates" and automatically determined Y-coordinates served as error measure.

3 Results

Supplementary Table 1 shows the approximate heights over the wooden plate and the distances from the camera for all POI. The distances in the rightmost column of

Supplementary Table 1 Points of interests' heights over base[1] and distances from camera.

model	point of interest	height over wooden plate in cm (h)	distance from camera in cm ($128 - h$)
fur-cov. m.	Isc.Tub., L	17.5	110.5
	Dish, L	16.1	111.9
	Tail	18.8	109.2
	Dish, R	16.3	111.7
	Is.Tub, R	17.7	110.3
plaster c.	Isc.Tub., L	12.3	115.7
	Dish, L	10.2	117.8
	Tail	13.5	114.5
	Dish, R	10.8	117.2
	Is.Tub, R	11.9	116.1

[1] The base was a wooden plate, and the camera was mounted 128 cm above the plate. The models were placed on the wooden plate for recording.

Supplementary Table 1 were used in Equation 3 to calculate the width in Y-direction of a pixel that has this distance from the camera.

Supplementary Table 2 presents the descriptive statistics of the errors between the "true Y-coordinates" calculated as described in section 2 and the Y-coordinates determined automatically by the software described in [1].

4 Discussion

No inferential statistics was done, because imprecisions could not be avoided in measuring the errors in automated Y-coordinate determination.

The descriptive statistics for the plaster cast implicate problems in precise Y-coordinate determination when the model was moving. Maximal and mean errors are strictly monotonically increasing from standstill to 30 cm/s, but the growths from 10 to 20 and 20 to 30 cm/s are significantly smaller than from standstill to motion. The analogies to the decreasing behavior of the plaster casts HQIratio (discussed in the main paper, Table 1) are recognizable. When it comes to the fur-covered model, maximal and mean errors are strictly monotonically decreasing from

Supplementary Table 2 Descriptive statistics of errors (in pixel) in automatically determined Y-coordinates.

	**fur-covered model**					**plaster cast**				
	left side			right side		left side			right side	
	Isc.Tub.	Dish	Tail	Dish	Isc.Tub.	Isc.Tub.	Dish	Tail	Dish	Isc.Tub.
**standstill**			N = 2138					N = 1875		
MAX	1	0	0	0	0	2	1	1	3	2
MIN	0	0	0	0	0	0	0	0	0	0
MEAN	0	0	0	0	0	0	0	0	0.29	0.10
ST	0.30	0	0.21	0.07	0.02	0.39	0.42	0.44	0.70	0.40
**10 cm/s**			N = 140					N = 360		
MAX	133	135	133	134	133	154	154	154	154	153
MIN	0	0	0	0	0	0	0	0	0	0
MEAN	54.89	55.47	54.76	55.21	55.28	42.54	42.45	42.18	42.57	42.62
ST	31.43	31.64	31.24	31.17	31.37	36.49	36.46	36.31	36.18	36.33
**20 cm/s**			N = 21					N = 305		
MAX	82	81	81	81	81	157	157	158	155	157
MIN	0	0	0	0	0	0	0	0	0	0
MEAN	22.24	21.29	21.90	21.57	21.43	60.27	60.93	60.14	58.43	58.94
ST	20.09	19.84	19.93	19.84	19.88	40.66	40.75	40.68	39.31	39.28
**30 cm/s**			N = 4					N = 230		
MAX	30	31	30	31	31	161	164	161	161	161
MIN	0	0	0	0	0	0	0	0	0	0
MEAN	14.76	14.75	14.5	14.25	14.25	63.33	63.08	63.19	63.84	63.58
ST	13.96	14.81	14.7	14.71	14.71	41.47	41.70	41.47	41.51	41.44

10 to 30 cm/s. As the number of high quality images recorded for the fur-covered model at 20 or 30 cm/s are so small, the descriptive statistics for the errors in Y-coordinate determination were difficult to interpret for those velocities. The "true Y-coordinates" had to be calculated from the starting values (i.e. the Y-coordinates in the first high quality image of each run). Thus, the number of non-zero errors gathered per run could at most be one less than the number of high quality images acquired during this run. No influence could be taken out of which run the remaining high quality images originate. Therefore, a small number of high quality images (as 21 for 20 cm/s or 4 for 30 cm/s) implies an even smaller number of non-zero errors, and limits the range of errors. The maximal and mean error values for 20 and 30 cm/s calculated for the fur-covered model are hence not meaningful. Even for 10 cm/s, the number of high quality images is much lower ($N = 140$) than the number of recorded high quality images for the plaster cast in motion ($N \geq 230$). Although the maximal error values of 133 are smaller than the corresponding maxima for the plaster cast (154), the mean error values are considerably larger (≈ 55 vs ≈ 42.5). This indicates a larger number of high error values, even though the errors are based on only half the amount of high quality images.

Author details
[1]Institute of Animal Breeding & Husbandry of Christian - Albrechts - University, Olshausenstraße 40, 24098 Kiel, Germany. [2]Institute for Agricultural Engineering & Animal Husbandry of Bavarian State Research Center for Agriculture, Prof.-Dürrwaechter-Platz 2, 85586 Poing-Grub, Germany.

References

1. Salau J, Haas J, Junge W, Bauer U, Harms J, Bieletzki S: **Feasibility of Automated Body Trait Determination using the SR4K Time-Of-Flight Camera in Cow Barns**. *Springer Plus* 2014, **3**(225). [Http://www.springerplus.com/ content/ 3/ 1/ 225].
2. Weber A, Salau J, Haas J, Junge W, Bauer U, Harms J, Suhr O, Schönrock K, RothfußH, Bieletzki S, Thaller G: **Estimation of Backfat Thickness Using Extracted Traits from an Automatic 3D Optical System in Lactating Holstein-Friesian Cows**. *Livestock Science* 2014, **165**(0):129–137, [[http://www.sciencedirect.com/ science/ article/ pii/ S1871141314001747]].
3. MESA-Imaging: *SR4000 User Manual, version 2.0*. http://www.mesa-imaging.ch/prodview4k.php. [Download: 18th of March 2013].
